# Insecticidal potential of *Lantana camara* L. ethanol and nano-silver extracts against the potato tuber moth (*Phthorimaea operculella* [Zeller])

**DOI:** 10.1186/s12870-026-08292-5

**Published:** 2026-02-10

**Authors:** Ayşe Yeşilayer, Erhan Gülsoy

**Affiliations:** Department of Plant Protection, Faculty of Agricultural, Tokat Gaziosmanpasa University, Tokat, Türkiye

**Keywords:** Biopesticides, *Lantana camara*, Silver nanoparticles (AgNPs), *Phthorimaea operculella*, Türkiye

## Abstract

The extensive reliance on synthetic pesticides has led to considerable ecological disruption and negative impacts on non-target organisms worldwide. As a sustainable alternative, nanotechnology offers promising avenues for the development of innovative and environmentally safe biopesticides. In this study, the insecticidal efficacy of ethanol and silver nanoparticles (AgNPs) extracts derived from *Lantana camara* L. was evaluated against the eggs and larvae of the Potato Tuber Moth (*Phthorimaea operculella* [Zeller, 1873]), a quarantine pest of major concern in potato production in Türkiye. Toxicological assessments revealed dose-dependent larvae mortality, with LC₅₀–LC₉₀ values ranging from 6.49–51.45 ppm for ethanol extracts and 1.47–5.88 ppm for AgNPs formulations. Maximum inhibition of egg hatching was observed at 12% ethanol extract (86.51%) and 200 ppm AgNPs treatment (94.95%). Bioassay results demonstrated that AgNPs formulations of *L. camara* were significantly more effective than ethanol extracts in suppressing both larval and egg development. Moreover, potato tubers treated with AgNPs exhibited a pronounced reduction in adult moth emergence compared to untreated controls. Collectively, these findings highlight the potential of *L. camara* nano-silver extracts as a sustainable biopesticide candidate for integrated management of *P. operculella*.

## Introduction

*Phthorimaea operculella* (Zeller, 1873) (Lepidoptera: Gelechiidae), commonly known as the potato tuber moth (PTM), is recognized as one of the most destructive pests of potato crops in warm climate regions, causing substantial yield losses and economic damage worldwide. The larvae feed on mesophyll tissue, producing blotch mines that later become necrotic, and may also penetrate stems and tubers, thereby reducing both crop quality and marketability [1, 2]. Conventional management strategies rely heavily on synthetic chemical insecticides,however, their extensive use has led to several challenges, including the development of insecticide resistance, environmental contamination, and adverse effects on non‑target organisms and human health [3, 4]. These limitations highlight the urgent need for safer and more sustainable alternatives in integrated pest management programs.

Biopesticides have emerged as promising eco‑friendly agents for pest control. Derived from natural sources, they offer advantages such as reduced toxicity, improved soil fertility, and minimal impact on biodiversity [5]. Recent studies emphasize that biopesticides not only lower chemical residues but also enhance resilience against pest resistance [6]. Nevertheless, despite their potential, many botanical extracts exhibit moderate efficacy, slower action, and limited persistence under field conditions, which restricts their widespread adoption [7, 8],). Similar limitations have been reported in the management of storage pests, including *Sitophilus oryzae* Hustache, 1930 (Coleoptera: Curculionidae) and *Tribolium castaneum* (Herbst 1797) (Coleoptera: Tenebrionidae), where crude plant extracts often fail to provide consistent protection under storage conditions, [3, 9]. In the case of *P. operculella*, the potato tuber moth, several studies have demonstrated that while botanical extracts such as *Lantana camara* L. (1753), exhibit larvicidal and ovicidal activity, their efficacy is concentration‑dependent and declines over time [4]. To overcome these limitations, nanotechnological formulations have gained increasing attention in agricultural pest management. Nanoparticles enable targeted delivery, enhance biodegradability, and sustain effective concentrations over extended periods, thereby reducing the required dosages and minimizing environmental contamination [10–12]. Recent findings confirm that green‑synthesized nanoparticles, particularly silver and silica‑based formulations, significantly improve insecticidal activity against both storage pests and *P. operculella*, offering a sustainable and potent alternative to conventional chemical pesticides [3, 13]. Among candidate plants,* L. camara*, widely cultivated in Turkey as an ornamental species [14], has demonstrated insecticidal properties attributed to its phytochemical constituents, including lantaden A and B [15–17]. Recent research has shown that green‑synthesized nanoparticles, particularly silver and zinc formulations, when combined with plant extracts, exhibit synergistic insecticidal activity and improved stability [3, 9, 13].

Taken together, these findings suggest that integrating *L. camara* extracts with green‑synthesized nanoparticles may provide a potent, environmentally sustainable, and cost‑effective alternative to conventional chemical pesticides. This study therefore aims to evaluate the larvicidal and ovicidal efficacy of ethanol and nano‑silver extracts of *L. camara* against PTM, thereby addressing the limitations of current pest management strategies and contributing to the development of innovative biopesticidal formulations*.*

### Experimental design and treatments

#### Plant material and insect culture

The immature developmental stages (larvae and eggs) of the PTM were used in this study. Potato tubers, obtained as the nutritional medium, were obtained from cultivation plots within the research fields of Tokat Gaziosmanpaşa University. Adult male and female specimens were maintained in 1‑liter plastic containers, with honey provided as a carbohydrate source. The container lids were replaced with sterile blotting paper secured by muslin cloth to allow ventilation while preventing escape. Oviposition by adult females occurred on the paper surface. The insect culture was maintained under controlled laboratory conditions (23 ± 2 °C, 65 ± 2% relative humidity, and a photoperiod of 12:12 h light:dark), consistent with protocols reported in previous bioassay studies on PTM [18].

The lantana plant materials used in this study were collected exclusively from wild populations. In accordance with national and institutional regulations, no specific permits or ethical approvals were required for collection. Leaves of *L. camara* were gathered during June and July from natural habitats in the provinces of Yalova and Mersin. Immediately after collection, samples were placed in labeled sterile paper bags, documenting locality and date of sampling, and transported under ambient conditions to the Entomology Laboratory of Tokat Gaziosmanpaşa University. Standardized preparation procedures were applied, including air‑drying under controlled environmental conditions (25 ± 2 °C, 60 ± 5% RH), to minimize microbial contamination and preserve morphological integrity. The processed material was stored in sealed containers until use in experimental assays, ensuring methodological consistency and reproducibility. Plant specimen identification was confirmed by Dr. Bederettin Selvi, Department of Biology, Tokat Gaziosmanpaşa University.

### Extract preparation and toxicity of lantana plant extract

Dried Lantana plant material was ground into a fine powder, and 20 g of the ground sample were extracted with 100 mL of ethanol. The mixture was agitated on a rotary shaker at 200 rpm for 24 h. Following extraction, the solution was filtered through sterile filter paper, and the filtrate was allowed to stabilize at room temperature. The resulting stock solution was subsequently diluted with ethanol to obtain the desired concentrations of 3, 6, 9, and 12% (w/v). Ethanol alone was used as the control treatment [19, 20].

### Green synthesis of silver nanoparticles (AgNPs)

**Fig. 1:**
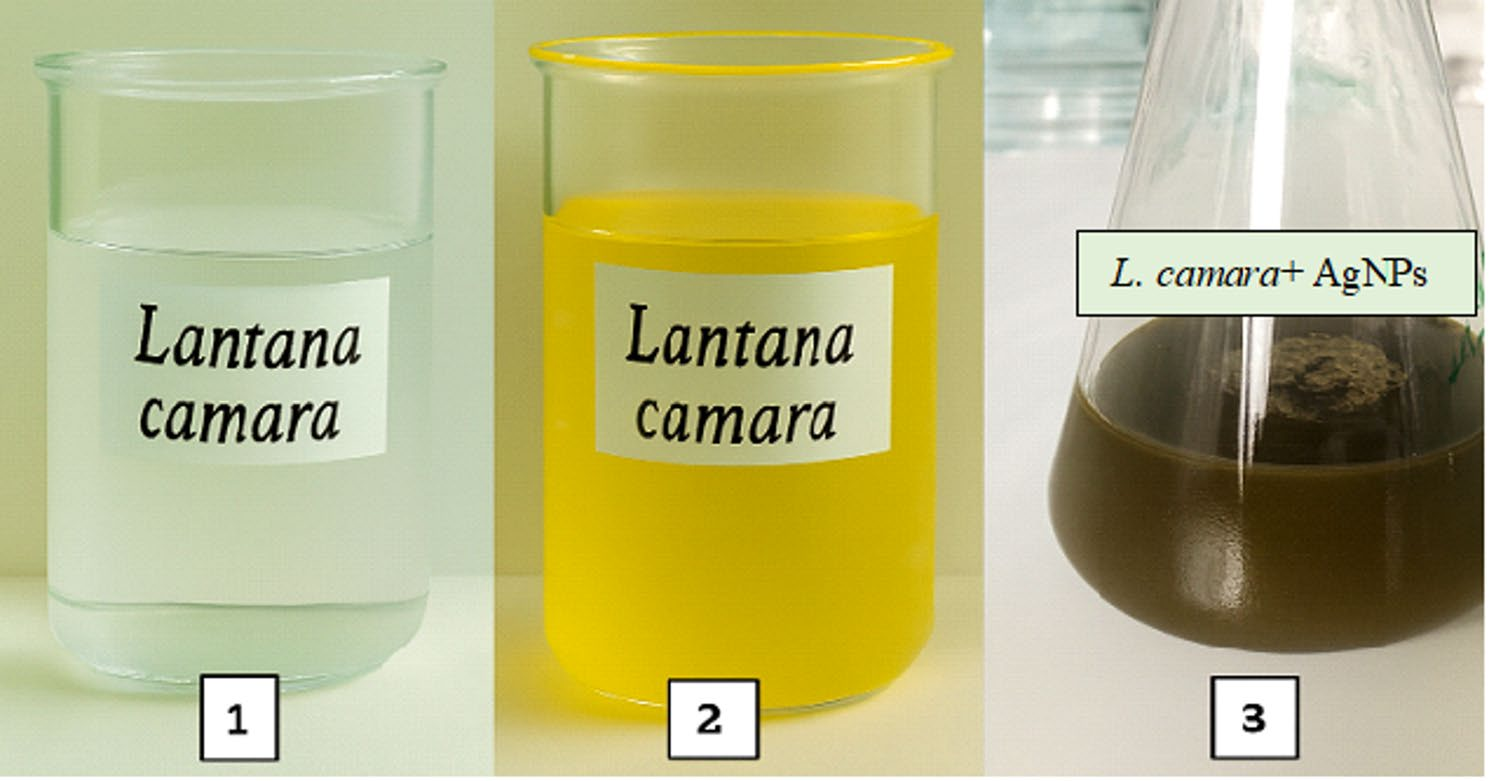
Green-synthesized nanoparticles changes (respectively 1, 2, 3) color after mixing of silver nitrate and *L. camara* extract

The green synthesis of silver nanoparticles (AgNPs) was carried out using a 6% concentration of *L. camara* extract, previously determined to induce over 50% larval mortality in bioefficacy assays. For the synthesis of 1 mM (≈168 ppm) AgNPs, 0.02 g of commercially sourced silver nitrate (AgNO₃; Nano-Kar) was dissolved in 100 mL of deionized water. The Lantana extract was then added to the silver nitrate solution. The mixture was incubated at 30 °C for 30 min, during which a visible color change from yellow to brown indicated the formation of silver nanoparticles (Fig. 1) [21]. For this synthesized solution, experimental concentrations were prepared: 0 ppm (control), 50, 100, 150 and 200 ppm [22–24].

### Toxicity assay on larvae

The larvicidal activity of the plant extract was assessed using 1–2-day-old larvae PTM. Potato tubers of uniform weight were immersed in extract solutions of varying concentrations for 3 to 5 s. Subsequently, the treated tubers were air-dried at room temperature for 15 to 20 min prior to larval exposure [25]. Dried potato tubers were placed into plastic containers measuring 5 cm in height and 11 cm in diameter. 10 larvae were carefully transferred onto each tuber using forceps. The containers were appropriately labeled and covered with transparent tulle to ensure aeration while preventing larval escape [26]. The number of live and dead larvae was recorded daily, and observations continued until the larvae progressed to pupal and adult stages. The experiment was conducted in two independent trials, each comprising *n* = 5 replications per treatment including a control, following standard bioassay protocols [27]. Since no significant differences were observed between trials, the data were pooled for statistical analysis, resulting in a total of 10 replicates per treatment. In the other part of the study, the dipping-slide method—previously employed for ethanol extract treatments—was utilized to assess the larvicidal effects of green-synthesized AgNPs derived from *L. camara*. A stock solution of AgNPs at 200 ppm was prepared and subsequently diluted to achieve the target concentrations for testing [28, 29]. The experiments were arranged in five replications, with ten larvae used per replicate. Control groups did not receive any treatment. Instead, ethanol was administered to the control group in the Lantana extract trials, whereas distilled water was used as the control in the AgNPs experiments. Larval mortality was recorded daily, and observations continued until the seventh day post-treatment. Each treatment was conducted in twice independent repetitions to ensure statistical robustness.

### Egg-hatching assay; inhibition with lantana extracts and with AgNPs

The inhibitory effects of *L. camara* and nano-silver extracts on egg hatching of PTM were evaluated under controlled laboratory conditions. For the plant extract assay, the dipping-slide method was employed following Oroumchi and Lorra [26]. Filter paper segments containing recently laid or one-day-old PTM eggs were cut into groups of twenty eggs each. These segments were immersed in the prepared extract solutions for approximately 3–5 s, air-dried, and transferred into sterile Petri dishes (90 mm). Each dish was sealed to maintain consistent humidity and temperature. Each treatment was performed with n = 5 replicates, including a control, and the experiment was independently repeated twice to ensure statistical validity. As the two independent trials yielded comparable outcomes without significant variation, their datasets were combined for statistical evaluation, providing a total of 10 replicates per treatment. In the *L. camara* extract assays, ethanol served as the control. Egg hatchability was monitored daily for seven days, and the numbers of hatched and unhatched eggs were recorded at the end of the observation period [30]. For the nano-silver assay, eggs were immersed in solutions of nano-silver extracts derived from *L. camara* at concentrations of 0, 50, 100, 150, and 200 ppm for 3–5 s. Following treatment, eggs were transferred into sterile Petri dishes under identical laboratory conditions. Each treatment included *n* = 5 replicates, with twenty eggs per replicate, and the experiment was independently repeated twice. Distilled water was used as the control in the AgNPs assays. The number of unhatched eggs was recorded at the end of the experimental period.

### Statistical analysis

The data were subjected to one-way analysis of variance (ANOVA), conducted independently for each observation day (3, 5, and 7 days) to account for temporal variation. Treatment means were compared at the *P* < 0.05 probability level, and differences among means were separated using Tukey’s HSD test. All statistical analyses were performed using Minitab (version 17, Minitab®). Mortality data were further analyzed by probit analysis following the method of Finney (1971) to estimate LC₅₀ and LC₉₀ values with their 95% confidence limits. The slope ± SE, chi-square (χ^2^), and heterogeneity values were calculated to evaluate the goodness-of-fit of the regression model. 

## Results

In the study conducted to evaluate the inhibition of egg hatching and larvicidal activity against PTM prior to the adult stage, both the ethanol extract of lantana and the aqueous extract of silver nanoparticles (AgNPs) were tested individually and in combination. The data obtained from these bioassays were comprehensively analyzed, and the differences among treatments were found to be statistically significant (*P* < 0.05), thereby confirming the efficacy of both extracts in reducing egg hatchability and larval survival.

### Efficiency of ethanol and nano-silver extracts of L. camara against PTM larvae

The results demonstrated that both ethanol and nano‑silver extracts of *L. camara* exhibited significant larvicidal activity against PTM larvae, with mortality rates increasing in a dose‑dependent manner over time. Ethanol extracts showed moderate efficacy, reaching the highest mortality at 12% concentration (77.69%) on the seventh day, which was statistically different from lower concentrations (*P* < 0.05). In contrast, nano‑silver extracts displayed a stronger and more rapid effect, with 200 ppm treatment causing 66.51% mortality as early as the third day and reaching 84.89% by the seventh day. Lower concentrations (50–150 ppm) also increased larval mortality but remained significantly less effective than 200 ppm. Mortality in the control groups remained negligible (< 0.2%), and the differences observed between treatments and controls were statistically significant (*P* < 0.05).

According to analysis of variance ANOVA revealed significant differences among treatments and statistical analysis (ANOVA followed by Tukey’s HSD test) confirmed that higher concentrations were significantly more effective than lower ones, with results on the 3rd days (*F* = 11.15, df = 4, *P* < 0.001 for ethanol; *F* = 18.99, df = 4, *P* < 0.001 for nano‑silver), 5th days s (*F* = 106.98, df = 4, *P* < 0.001 for ethanol; *F* = 94.72, df = 4, *P* < 0.001 for nano‑silver) indicating that both concentration and exposure time had a pronounced effect on larval mortality (Table 1).

**Table 1:** Effect of ethanol (%) and nano‑silver (ppm) extracts of *L. camara* on the larvicidal activity against *P. operculella* larvae on 3, 5, and 7 days (mean ± SE)

Ethanol extracts (%)	3rd day	5th day	7th day	Nano-silver extracts (ppm)	3rd day	5th day	7th day
3%	9.83 ± 2.01 c	34.73 ± 0.34 d	36.12 ± 0.21 d	50 ppm	10.79 ± 0.81 c	37.39 ± 0.19 c	39.89 ± 0.21 bc
6%	14.64 ± 0.20 cd	41.13 ± 0.36 c	52.37 ± 0.3 c	100 ppm	18.35 ± 0.25 bc	42.37 ± 0.39 bc	55.33 ± 0.31 b
9%	16.65 ± 0.57 b	52.52 ± 0.18 b	63.33 ± 0.20 b	150 ppm	23.59 ± 0.13 b	52.57 ± 0.23 b	70.25 ± 0.25 ab
12%	25.12 ± 0.95 a	62.61 ± 0.19 a	77.69 ± 0.21 a	200 ppm	66.51 ± 0.34 a	75.45 ± 0.32 a	84.89 ± 1.24 a
Control	0.00 ± 0.00 d	0.00 ± 0.00 e	0.16 ± 0.46 e	Control	0.16 ± 0.46 d	0.16 ± 0.46 d	0.16 ± 0.46 d

^*^Means followed by different letters within the same column (day of observation) are significantly different according to Tukey’s HSD test (*P* < 0.05)

### Efficiency of ethanol and nano-silver extracts of L. camara against PTM Eggs

At a 6% concentration of ethanol extract from *L. camara*, the egg‑hatching inhibition rate of PTM was recorded as 60.69%, whereas a 100 ppm concentration of nano‑silver extract achieved a slightly higher inhibition rate of 68.82%. In the control (deinozed water) group, nearly all eggs successfully hatched. The maximum inhibition was observed at the highest concentration of nano‑silver extract (200 ppm), reaching 94.95%, while ethanol extract at 12% concentration resulted in 86.51%. In both treatment types, the inhibition rate increased proportionally with concentration ant depending on time. Statistical analysis revealed significant differences among treatments on 7 th day (*F* = 123.13, df = 4, *P* < 0.001 for ethanol extracts; *F* = 110.77, df = 4, *P* < 0.001 for nano‑silver with lantana extracts), was indicated that concentration had a pronounced effect on egg‑hatching inhibition. The highest concentrations (12% ethanol and 200 ppm nano‑silver) were statistically different from lower concentrations (*P* < 0.05). On the whole, nano‑silver extracts exhibited a stronger inhibitory effect compared to ethanol extracts, particularly at higher concentrations (Table 2).

**Table 2:** Toxicity effects of ethanol and nano-silver extracts of *L. camara* on *P. operculella* eggs (mean ± SE)*

Treatment (%)	*Lantana camara* ethanol extracts	Treatment (ppm)	*Lantana camara* nano-silver extracts
3%	46.87 ± 0.05 d	50 ppm	54.39 ± 0.06 d
6%	60.69 ± 0.12 c	100 ppm	68.82 ± 0.08 c
9%	73.82 ± 0.06 b	150 ppm	80.77 ± 0.10 b
12%	86.51 ± 0.34 a	200 ppm	94.95 ± 0.80 a
Control	0.08 ± 0.02 e	Control	0.08 ± 0.02 e

^*^ Means followed by different letters within the same column are significantly different according to Tukey’s HSD test (*P* < 0.05)

### *Lethal concentration (LC) analysis of* Lantana camara *ethanol and nano-silver extracts against PTM larvae and egg*

#### Larvae

Following these observations, lethal concentration (LC) values were estimated over a seven‑day observation period to provide a more precise quantification of the toxic effects of ethanol and nano‑silver extracts. This analysis allowed for the determination of LC₅₀ and LC₉₀ values, thereby enabling a direct comparison of their relative potency and concentration–response relationships (Table 3 and Table 4).

**Table 3:** Estimated lethal concentration (LC₅₀ and LC₉₀) values, slope ± SE, χ^2^, and heterogeneity for ethanol extracts of *L. camara* and their combination with AgNPs against *P. operculella* larvae

Treatment	LC_50_ (95% CL)	LC _90_ (95% CL)	Slope ± SE	χ^2^	Heterogeneity
*L. camara*	6.49 (5.57–7.52)	51.45 (32.40–115.13)	1.42 ± 0.20	18.51	0.24
*L. camara* + Ag-Np	1.47 (1.28—1.64)	5.88 (4.84–7.77)	2.13 ± 0.21	31.66	0.41

LC values are expressed as % and ppm, estimated from larval bioassays conducted over seven days

**Table 4:** Estimated lethal concentration (LC₅₀ and LC₉₀) values, slope ± SE, χ^2^, and heterogeneity for ethanol extracts of *L. camara* and their combination with AgNPs against *P. operculella* eggs

Treatment	LC_50_ (95% CL)	LC_90_ (95% CL)	Slope ± SE	χ^2^	Heterogeneity
*L. camara*	3.96 (2.81—4.87)1	40.96 (24.76—113.33)	1.26 ± 0.22	7.65	0.25
*L. camara* + Ag-Np	1.71 (1.31–2.07)	20.05 (10.84—73.51)	1.19 ± 0.22	10.96	0.33

LC values are expressed as % and ppm, estimated from egg-hatching bioassays conducted over seven days

The insecticidal efficacy of the ethanolic extract of *L. camara*, both individually and in combination with (AgNPs, against PTM larvae is detailed in Table 3. Probit analysis of the mortality data revealed a significant enhancement in toxicity when the *L. camara* extract was formulated with AgNPs.

The median lethal concentration (LC₅₀) for the *L. camara* extract alone was 6.49 ppm, whereas the combined formulation with AgNPs exhibited a markedly lower LC₅₀ of 1.47 ppm, corresponding to a 4.4‑fold increase in toxicity. A similar trend was observed for LC₉₀ values, which declined from 51.45 ppm for the extract alone to 5.88 ppm for the nano‑formulation, indicating that the combination treatment was approximately 8.75 times more effective in achieving 90% mortality. The non‑overlapping 95% confidence limits for both LC₅₀ and LC₉₀ confirm a statistically significant difference in potency between treatments. Moreover, the steeper slope of the dose–response curve for *L. camara* + AgNPs (2.13 ± 0.21) compared to the extract alone (1.42 ± 0.20) suggests a more rapid mortality response with increasing concentrations. Finally, the chi‑square (χ^2^) values for both treatments demonstrated a good fit to the probit model, supporting the robustness of the analysis.In summary, these results demonstrate a potent synergistic effect, where the integration of AgNPs significantly enhances the insecticidal properties of the *L. camara* extract against PTM larvae under the specified experimental conditions.

#### Egg

Probit analysis demonstrated that nano‑silver formulations of *L. camara* exhibited substantially greater ovicidal potency than the ethanol extract alone. The LC₅₀ value for the ethanol extract was 3.96% (95% CL: 2.81–4.87), whereas incorporation of silver nanoparticles (AgNPs) reduced the LC₅₀ to 1.71 ppm (95% CL: 1.31–2.07), indicating a marked enhancement in ovicidal activity. A similar trend was observed for LC₉₀ values, which declined from 40.96% (95% CL: 24.76–113.33) for the ethanol extract to 20.05 ppm (95% CL: 10.84–73.51) for the AgNPs formulation, reflecting increased effectiveness at higher exposure levels. These findings clearly indicate that silver nanoparticle incorporation significantly amplifies the bioactivity of *L. camara* extracts by lowering the lethal concentrations required to inhibit egg hatching (Table 4).

The relatively low slope values (1.26 ± 0.22 for ethanol; 1.19 ± 0.22 for AgNPs) suggest a shallow dose–response relationship, likely reflecting heterogeneity in egg susceptibility or variability in embryonic physiological conditions. Such shallow slopes are commonly reported in ovicidal bioassays, where factors such as chorion thickness, embryonic developmental stage, and micro‑environmental conditions contribute to non‑uniform responses. Nevertheless, the χ^2^ values (7.65 and 10.96) and heterogeneity indices (0.25 and 0.33) indicate an acceptable fit of the probit model to the observed data, supporting the robustness and reliability of the estimated lethal concentrations. In terms of the mode of action, the enhanced ovicidal efficacy of AgNPs may be attributed to their nanoscale size, which facilitates penetration through the egg chorion and disrupts embryogenesis via oxidative stress induction, dehydration, and impairment of cellular integrity. This nanoparticle‑mediated action likely acts synergistically with the phytochemical toxicity of *L. camara*, which is rich in bioactive compounds such as alkaloids, flavonoids, and terpenoids known to interfere with insect growth and reproductive processes. The combined effects of silver nanoparticles and plant secondary metabolites plausibly account for the pronounced reduction in LC values and narrower confidence intervals observed in the nano‑formulated treatment.

## Discussion

This study examined the larvicidal and ovicidal effects of ethanol and green‑synthesized nano‑silver extracts of *L. camara* on the immature stages (eggs and larvae) of PTM. Silver nanoparticles were synthesized via a green method, utilizing *L. camara* extracts as a reducing agent, due to the method’s enhanced reliability and environmental compatibility [31]. Larval mortality began to manifest by the third day across all concentrations of ethanol extracts, with a mortality rate 52.37% recorded at a 6% concentration by day seven. Similarly, Kasmara et al. [32] reported mortality rates exceeding 55% in *Spodoptera litura* (Fabricius 1775) (Lepidoptera: Noctuidae) larvae treated with *L. camara* extracts at concentrations ranging from 5 to 40%. Pavela [27], investigating methanol extracts from various plant species against *Spodoptera littoralis* Boisduval, 1833 (Lepidoptera: Noctuidae) larvae, found that several—including *Foeniculum vulgare* Mill. 1768 and *Artemisia campestris* L. 1753, —achieved 100% mortality by the fifth day, while others such as *Lavandula angustifolia* Mill., 1768 and *Artemisia absinthium* L., 1753 induced 39% mortality. In our study, the highest ethanol extract concentration resulted in 62.6% larval mortality by day five.

In comparison, the AgNPs extract of Lantana demonstrated enhanced toxicity, with larval mortality rates of 75.45% and 84.89% on the fifth and seventh days, respectively. The LC₅₀ and LC₉₀ values for ethanol extracts were calculated at 6.49% and 51.45 ppm, respectively, while those for nano‑silver extracts were markedly lower, at 1.47 and 5.88 ppm, indicating higher potency. Supporting findings by Kasmara et al. [32] demonstrated increased larvicidal activity of nano‑formulated *L*. *camara* compared to crude extracts on third instar *S. litura* larvae. Furthermore, earlier studies have indicated that silver nanoparticles can induce larval mortality by penetrating the cuticle and causing dehydration and cellular disruption [33]. More recent work by Alghamdi & Basher [34], confirmed the larvicidal efficacy of *L. camara* ethanol extracts against *Anopheles arabiensis* Patton, 1905 (Diptera: Culicidae) and *Culex quinquefasciatus* Say, 1823 (Diptera: Culicidae) larvae, mosquito larvae, with LC₅₀ values in the range of 5–7% and LC₉₀ values exceeding 50%, closely matching our ethanol extract results, researcher are who attributed the enhanced toxicity of silver nanoparticles to their ability to penetrate the larval cuticle and disrupt cellular integrity, while Ratan et al. [35] demonstrated that green‑synthesized AgNPs from *L. camara* leaves exhibit strong antimicrobial and bioactive properties.

The ovicidal potential of both ethanol (3–12%) and nano‑silver (50–200 ppm) *Lantana* extracts was also evaluated. Egg‑hatching inhibition at the lowest and highest concentrations ranged from 46.87 to 86.51% for ethanol and from 54.39 to 94.95% for nano‑silver treatments. Both treatments exhibited significantly greater ovicidal activity than the control group. Khani et al. [36] reported inhibition rates of 59 and 58% at the lowest doses of petroleum ether extracts of *Piper nigrum* L., 1753 and *Jatropha curcas* L., 1753, respectively. Similar ovicidal effects were also documented using *Allium sativum* L., 1753 and *Curcuma longa* L., 1753 against *T. castaneum*. In a comparative study, Elsayed [37] found that *L. camara* and *Solanum nigrum* L., 1753 extracts both inhibited egg hatching, with *S. nigrum* showing higher efficacy. Additionally, Gülsoy & Yeşilayer [38], demonstrated that *L. camara* extracts inhibited *P. operculella* egg hatching by 20% more than *Salvia* spp., a finding reinforced by Yeşilayer & Gülsoy [38]. Yeşilayer & Deniz [39] reported that extracts of *Salvia officinalis* L., 1753 achieved egg‑hatching inhibition rates of 52.5 at 3% and 62.5 at 5%, with *Thymus vulgaris* L., 1753 and *Lavandula angustifolia* Mill., 1768 reaching up to 73.75 at 10% concentration. Consistent with larvicidal outcomes, nano‑silver *L. camara* extracts were more effective in inhibiting egg hatching, with LC₅₀ and LC₉₀ values of 3.96 ppm and 40.96 ppm, respectively.

## Conclusion

In the context of integrated pest management (IPM), there is an increasing emphasis on the development of green nanobiotechnological solutions that are both efficient and environmentally benign. Nanotechnology has progressively found applications in agricultural and agronomic practices [40, 41]). The present study demonstrates the promising insecticidal potential of both ethanol and nano-silver *L. camara* extracts against the pre-imaginal stages of PTM. As this is the first study to document the pesticidal activity of nano-silver *Lantana* extract against PTM, further investigations under field and semi-field conditions are warranted to validate its stability and efficacy.

Overall, the findings clearly indicate that nano-silver *L. camara* extracts exert superior larvicidal and ovicidal activities compared to ethanol-based formulations. Therefore, both ethanol and nano-silver *L. camara* extracts could serve as effective and sustainable alternatives to synthetic pesticides in the management of PTM and other agricultural pests.

## Data Availability

All data generated or analyzed during this study are included in this published article. Additional datasets are available from the corresponding author upon reasonable request.

## Abbreviations

AgNPs: Silver nanoparticles
PTM: *Phthorimaea operculella*
